# Amorphous RuCoP Ultrafine Nanoparticles Supported on Carbon as Efficient Catalysts for Hydrogenation of Adipic Acid to 1,6-Hexanediol

**DOI:** 10.3390/ma15228084

**Published:** 2022-11-15

**Authors:** Wei Gong, Xuyun Wang, Shan Ji, Hui Wang

**Affiliations:** 1 State Key Laboratory Base for Eco-Chemical Engineering, College of Chemical Engineering, Qingdao University of Science and Technology, Qingdao 266042, China; 2 College of Biological, Chemical Science and Chemical Engineering, Jiaxing University, Jiaxing 314001, China

**Keywords:** adipic acid, catalyst, 1,6-hexandiol, hydrogenation, RuCoP

## Abstract

As an important raw material for organic synthesis, the 1,6-hexanediol (HDOL) is synthesized by the complicated two-step process traditionally. The hydrogenation of adipic acid (AA) is a potential way to prepare 1,6-hexanediol. At present, amorphous RuMP (M: Co, Ni, Fe, etc.)-based alloys with low Ru content were developed by co-precipitation as the efficient catalysts for converting AA to HDOL via hydrogenation. Among these RuMP catalysts, RuCoP alloys exhibited the highest selectivity and yield to HDOL owing to the electronic effect. The selectivity and yield of HDOL for the optimized RuCoP/C sample was achieved to 80% and 64%, respectively, at 65 bar and 220 °C. A series of RuCoP alloys with different degrees of crystallinity and particle sizes were prepared to investigate the effect of morphology and structure on its catalytic performance. The results indicated that the high catalytic activity of RuCoP/C resulted from its rich active sites due to its amorphous phase and small particle size.

## 1. Introduction

The 1,6-hexanediol (HDOL) is an important chemical, which has been widely used as a precursor to produce polyurethanes and coatings. It is expected that the global market of HDOL will increase from USD 728 million in 2016 to USD 1,042 million in 2021 [1]. Usually, a two-step procedure is applied to produce HDOL from adipic acid (AA), namely, (1) the esterification of AA and methanol for preparing dimethyl adipate (DMA); (2) DMA is then hydrogenated to form HDOL [2,3]. Unfortunately, this two-step procedure is a high energy-consumption process, making the whole procedure of low economical value for commercialization. In order to solve these problems, a more direct production of HDOL via hydrogenating AA was proposed, and many efforts have been contributed to develop catalysts with high selective and catalytic abilities [4,5]. However, the proportion of precious metals in the preparation of catalysts is too high, and the proportion of precious metals is large. Therefore, it is urgent to find a catalyst with a small proportion of precious metal and high conversion and yield.

Extensive research indicated that Ru was an active component in the catalysts for the hydrogenation transformations. Additionally, the higher catalytic activity can be achieved by alloying Ru with other metals and loading Ru onto the supports with high surface and thermal stability [6,7,8,9]. For instance, Ru was alloyed with other metals such as Pt [10,11,12], Sn [13], and Zn to obtain good selectivity for the hydrogenation of, for example, oleic acid toward fatty alcohols owing to the modification of the electronic structure of the Ru atom. Ru, Rh, Ni, Ni, Cr, or another metal is used to catalyst adipic acid. [14,15]. Additionally, amorphous Ru/Nb_2_O_5_ is highly active in the hydrogenation reaction [16]. The hydrogenation of Ru with other particles has been thoroughly investigated by some people [17,18,19]. Although significant progress has been made in the development of Ru-based catalysts for the hydrogenation reaction, the high cost and scarcity of Ru impeded its large-scale application as hydrogenation catalysts.

Amorphous alloys have received much attention of late owing to its more active sites on their surface resulting from their short-range order and long-range disorder structure [20,21,22]. Additionally, amorphous Ru alloys have demonstrated outstanding catalytic activity for hydrogenation reactions [23,24]. Many amorphous noble-metal-based alloy catalysts have been developed for hydrogenation reaction, such as RuB amorphous alloys, which were used for the benzene-selective hydrogenation to cyclohexene [25,26]; a NiPdB-PEG(800) amorphous alloy catalyst, which was employed for the chemoselective hydrogenation of electron-deficient aromatic substrates [27,28]; and RuSnB/TiO_2,_ which was used as the selective catalyst for the hydrogenation of fatty acid to fatty alcohols [29]. Introducing transition metals into the amorphous catalyst could suppress the growth of amorphous particles. Additionally, amorphous catalysts possess higher electron density, which leads to higher activity in the hydrogenation reaction [30,31]. However, there are few reports on the hydrogenation of AA to HDOL over amorphous Ru-based alloy catalysts [32,33].

In this work, the amorphous RuCoP alloy supported on commercial carbon was prepared by co-precipitation and used as a catalyst for the hydrogenation of AA to HDOL. The electronic effect of transition metals (Fe, Co Ni, Zn, and Cu) on the catalytic activity of amorphous Ru alloys was studied. The results found that the optimized RuCoP catalyst exhibited highly catalytic performance for the hydrogenation of AA to HDOL due to the large number of active sites available on its surface.

## 2. Experimental

### 2.1. Catalyst Preparation

RuCoP was prepared by NaBH_4_ reduction by co-precipitation. The detailed preparation process can be described as follows.

In total, 81 mg of CoCl_2_∙H_2_O, 151 mg of NaH_2_PO_2_∙H_2_O, and 14 mg RuCl_3_∙2H_2_O were dissolved in 30 mL of water with N_2_ bubbling through the solution throughout the experiment. Pre-treated carbon black Vulcan XC-72R (45 mg) (Carbot Corp., Boston, MA, USA) was added to the mixture with magnetic stirring. Afterward, 0.2 mol l^−1^ NaBH_4_ (20 mL) aqueous solution was added slowly to the mixture and then kept stirring for 1 h. The pH of the aqueous was adjusted to 8 by NaOH solution (6 mol L^−1^). The obtained precipitate was filtrated out and washed with deionized water several times. The washed product was kept in an ethanol solution. The atomic ratio of Ru:Co:P in precursors was 1:7:20. RuCo_7_P_20_/C catalysts with different crystallinity were prepared by heating at various temperatures, namely, 200, 300, and 400 °C, for 2 h under N_2_ atmosphere. The obtained samples were labeled as RuCoP/C-200, RuCoP/C-300, and RuCoP/C-400, respectively. For comparison, the numerous catalysts that are presented in Table 1 were also prepared according to the bubble template method.

### 2.2. Catalytic Performance

The amorphous RuCoP/C catalyst was used as a catalyst for the hydro-generation of AA to HDOL, which was carried out in a batch reactor (miniature autoclave JWCGF-H100 mL, TKA, Xian, China). In total, 0.2 g of AA, 60 mL of H_2_O, and 0.1 g of amorphous catalyst were added into the reactor. N_2_ was applied to flush the reactor three times and followed by purging with H_2_ stream (100 mL min^−1^, for 10 min) three times. The reactor was then pressurized to 30 bar by H_2_ and heated to 220 °C. At 220 °C, the pressure in the reactor reached 65 bar. Once the temperature was 220 °C, the time was set as zero, and the stirring system started to work with a rotating rate of 250 rpm. After the reaction reached certain times, the reactor was immediately immersed in ice water. For the recycling test, each run was performed for 6 h. Every time, the recycled catalyst was washed with deionized water three times and dried at 60°C overnight. The composition of the final products was analyzed by gas chromatograph (GC, Shimadzu GC-14, Shimadzu, Suzhou, China).

AA conversion (X) and HDO selectivity (Si) were calculated using the following the equations:

X = (mole of AA) in − (mole of AA) out/(mole of AA) in × 100%(1)

Si = (moles of product i)/(the sum of moles of products) × 100%(2)

Yi = (moles of product i)/((mole of AA) in ) × 100%(3)

### 2.3. Characterization

X-ray diffraction (XRD) spectra of the as-prepared catalysts were recorded on a Shimadzu XD-3A (Shimadzu, Japan) X-ray diffractometer using a filtered Cu Kα radiation (λ = 0.15418 nm) generated at 40 kV and 30 mA. Transmission electron microscopy (TEM) images were captured on a JEOL (JEM-2000 FX, JEOL Corporation, Tokyo) microscope operating at 200 kV. The Brunauer–Emmett–Teller (BET) method was employed to determine specific surface areas for obtained carbon materials, and the pore size distribution was calculated by a density functional theory (DFT) method using a slit pore NLDFT equilibrium model on a Quantachrome Autosorb-1 volumetric analyzer (Autosorb-iQ, U.S.). The temperature-programmed reduction with hydrogen (H_2_-TPR) was carried out using a purified mixture of H_2_/He (10/90 vol%) serving as a simultaneous carrier and reducing gas at a total flow rate of 40 mL min^−1^. Before the measurements, a catalyst sample (45 mg) was preheated in a dry He stream at 350 °C for 30 min. Afterwards, the sample was cooled down to 45 °C and the TPR experiment was initialized. The sample was heated at a rate of 10 °C min^−1^ to a final temperature of 600 °C. X-ray photoelectron (XPS) spectra were generated using a VG Escalab210 spectrometer fitted with a Mg 300 W X-ray source (VG, UK).

## 3. Results and Discussion

**Figure 1:**
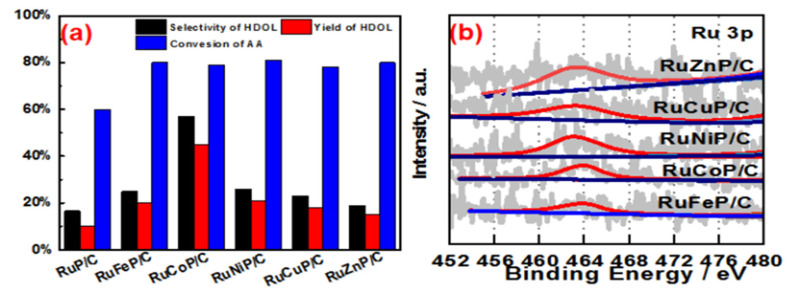
(**a**) Hydrogenating AA to HDOL on the different metal-doped amorphous alloy catalysts at 220 °C with a H_2_ pressure of 65 bar. (**b**) Ru 3p3/2 XPS spectra of the different metal-doped alloys.

The substitution of rare-earth metals for precious metals has always been the core topic of synthesis methods. 3d transition metals exhibit different chemical properties than 4d or 5d transition metals. Smaller static positions, lower electronegativity, and the potential involvement of one-electron processes provide the first-row transition metals with unique properties in related catalysis [34]. Hence, the first-row transition metals (M), including Fe, Co, Ni, Cu, and Zn, were selected to alloy with Ru in this work because the electronic effect between Ru and the transition metals has been demonstrated to promote the catalytic activity of Ru [35]. Here, a series of transition metal-doped RuMP (M = Fe, Co, Ni, Cu, and Zn) alloys was prepared and used for catalyzing the hydrogenation of AA. As shown in Figure 1, all as-prepared RuMP catalysts exhibited good catalytic performance for the hydrogenation of AA verified by 60~80% of conversion of AA. Among all the doped RuMP catalysts, RuCoP catalysts demonstrated the best selectivity (80%), indicating RuCoP alloys were the best catalyst for hydrogenation of AA to HDOL.

It should be noted that the temperature and pressure of the conversion from AA to HDOL were optimized by using RuCoP alloy as the catalysts, as displayed in Figure S1. The effect of reaction temperature and pressure on catalytic performance were also investigated and shown in Figure S1. 

The products include HDOL, cyclopentane, and others. Figure S1a shows that the catalytic activity increased slightly (the conversion rate increased from 85% to 90%) with the reaction temperature. The selectivity to HDOL obviously increased with the temperature. When the temperature was 220 °C, the selectivity to HDOL reached a peak number, namely, 65%. After that, the selectivity to HDOL started to drop due to the C-C or C-O cracking in AA at high temperature and resulted in the low selectivity. As shown in Figure S1b, the conversion of AA increased from 69% to 88% when the reaction pressure changed from 30 to 70 bar. The selectivity to HDOL was also increased with the pressure. Therefore, the optimized temperature and pressure for the hydrogeneration of AA to HDOL are 220 °C and 65 bar, respectively.

**Figure 2:**
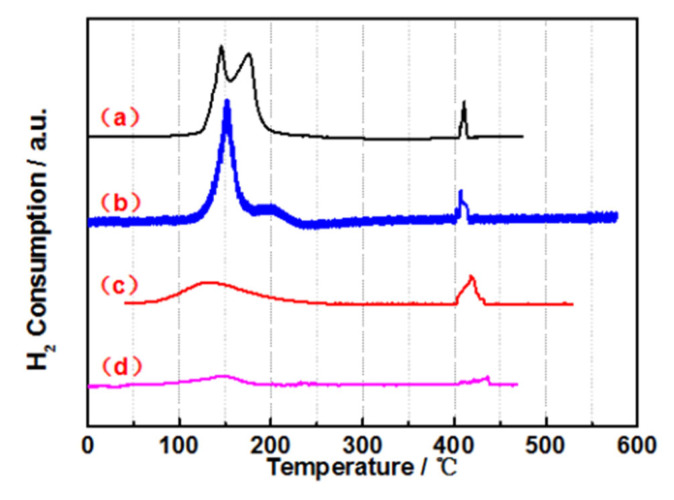
H_2_-TPR spectra of the amorphous alloy catalysts: (a) Ru1.0Co7P20/C, (b) Ru1.0Co17.5P20/C, (c) Ru1.0Co17.5P33.5/C and (d) Ru0.2Co7P20/C.

H_2_-TPR was performed, and the results are shown in Figure 2. It is clear that three main peaks were observed; the peaks at 150~200 °C were attributed to the reduction in the Ru species, and the peak at 400~450 °C was assigned to the reduction of Co(+2) to Co(0). The enhancement of the characteristic peak from Ru gradually increased, as shown in Figure S2. The peak area decreases as the amount of Co and P components increases. The reason for this phenomenon might be due to the appearance of oxidized cobalt and phosphorus, which increase the distance between the adjacent Ru atoms, leading to a decrease in hydrogen absorption capacity. The Ru1.0Co7P20/C with high catalytic activity has the best reduction capacity and the largest peak area. Apparently, Ru1.0Co7P20/C-derived catalysts had better dispersions of Ru than others. A series of catalysts of RuCoP/C, an outstanding amorphous phase, and a small particle size were achieved by Ru1.0Co7P20/C.

To reveal the electronic effect of these transition metals on the electronic structure of Ru and P, X-ray photoelectron (XPS) analysis for RuMP alloys was carried out. XPS survey spectra provided in Figure S2a indicated each type of alloy has the Ru and P elements besides the different transition metals. The Ru 3p XPS of all the alloys was fitted into two pairs of doublets, as shown in Figure S3c–g. The peak at ~462 eV is attributed to Ru(0), while the two peaks at ~464 eV in all catalysts are assigned to anhydrous Ru(+4). The P 2p XPS of all the alloys was fitted into one pair of doublets, as shown in Figure S2c–g. The peak at ~133.5 eV is attributed to P, which is oxidized by exposure to air. As shown in Table S1, the Ru in all the alloys is shown in the metallic state and oxidation state, and the metallic Ru species constituted half of the total Ru atoms. It is obvious in Figure 1b and Figure S2b that the binding energies of Ru 3d and P 2p show positive shifts from RuZnP/C to RuFeP/C. In particular, the highest binding energy shift of Ru 3p XPS in RuCoP/C refers to the downshift of the center of the d-band in respect to the Fermi level, which would lead to the decrease in the bond strength of adsorbents towards small molecules formed during the catalysis, resulting in high catalytic activity. The result implies that Ru and the transition metal element Co form the best electron cloud effect, which is conducive to the hydrogenation process of C=O.

In order to improve the cost performance of the RuCoP/C catalyst, its composition was optimized. When the Ru content was increased from 1 wt% to 5 wt%, the yield of HDOL gradually increased (17% to 61%), and the selectivity increased from 16.6% to 80% (shown in Table 1), indicating an obvious influence of Ru content on the performance of the catalyst. However, the conversion and selectivity showed no significant increase when the content of Ru increased to 6 wt%, suggesting that 5 wt% is the optimized amount for Ru in the RuCoP catalyst. As expected, the amount of Co and P in RuCoP also has a significant impact on the selectivity and conversion. As listed in Table 1, with the increased amount of Co and P in RuCoP, the yield of HDOL increased first and then decreased. When the amount of Co and P was 20 wt% and 30 wt%, respectively, the conversion of AA was about 80% and the yield of HDOL reached 64%. Therefore, the RuCoP/C catalyst with the Ru:Co:P atomic ratio of 1:7:20 was adopted for the all of the following experiments, which show the best electron cloud effect.

**Figure 3:**
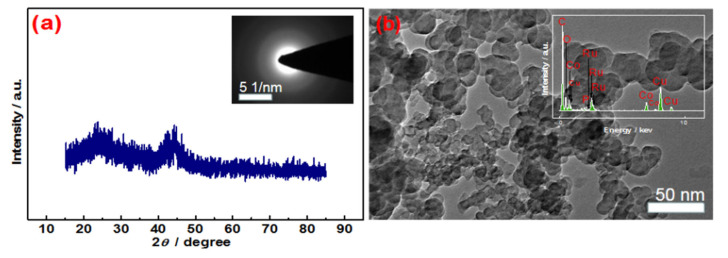
XRD patterns (**a**), TEM images (**b**) of RuCoP/C; insert: SAED pattern (**a**), and EDX (**b**).

The physical feature of the optimized RuCoP/C sample was characterized for revealing the origin of its catalytic activity. A broad peak at ca. 2θ = 45° was observed in the XRD pattern of fresh RuCoP/C catalyst except for the graphite peak of carbon (2θ = 26°) (Figure 3a), suggesting its amorphous structure [36], which was also confirmed by a halo ring in the selected area electron diffraction (SAED) image (Inset of Figure 3a). In the TEM image of RuCoP/C (Figure 3b), the particles with an average size of ca. 50 nm were the typical morphology of the commercial carbon support. EDX (Inset of Figure 3b) exhibited that there are Ru, Co, and P atoms in these particles. In the zoom-in TEM image (Figure S4a), small nanoparticles with a size of ca. 1 nm were found. However, clear lattice fringes cannot be observed in Figure S3b, suggesting the existence of amorphous phase. The XRD and TEM results reveal that the obtained RuCoP nanoparticles formed on carbon are ultrasmall and amorphous. Based on the above results, an amorphous structure and a small particle size were attributed to the catalytic performance of RuCoP/C. Herein, pretreated activated carbon is used as a typical support to understand the role of the Ru/Co/P atomic ratio in promoting the chemical and surface properties as well as the catalytic behavior [37].

**Figure 4:**
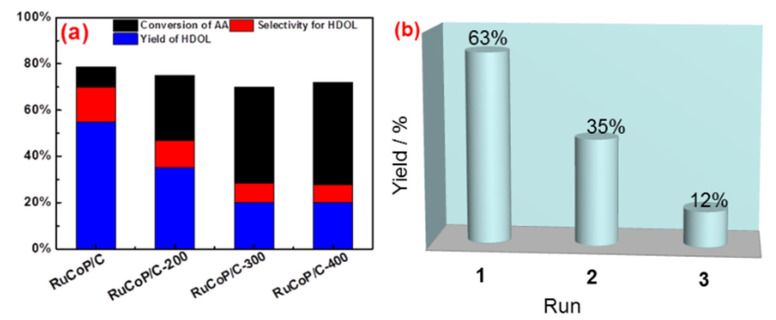
Hydrogenation of AA to HDOL on RuCoP/C, RuCoP/C-200, RuCoP/C-300, and RuCoP/C-400 (**a**); stability of the amorphous RuCoP/C catalyst in hydrogenation of AA to HDOL (**b**).

To confirm the above point, RuCoP/C was treated at 200, 300, and 400 °C, leading to the change of the crystal structure and particle size. As shown in Figure S4a, the amorphous state of RuCoP/C was transformed into the crystalline structure when the temperature was 400 °C. Meanwhile, as shown in the TEM images in Figure S4b–d, RuCoP nanoparticle aggregation was found with the increase in temperature. The above results indicated that the temperature causes the RuCoP nanoparticles to become a crystal structure and particle size to increase, leading to the decrease in the specific surface area (Figure S5). As exhibited in Figure 4a, the conversion of AA to HDOL of the RuCoP/C sample decreased and the selectivity to HDOL decreased from 70% to 30% when the temperature increased from room temperature to 400 °C (the yield of HDOL 58% to 20%). Therefore, the decrease in the catalytic performance of the RuCoP/C samples was attributed to the decrease in the active sites resulting from the transition of the amorphous structure and the particle aggregation, as well as the decrease in the specific surface area during the heat-treatment process.

The stability of amorphous RuCoP/C in the hydrogenation reaction of AA is investigated and shown in Figure 4b. In the first batch, the yield of HDOL is 63%. The yield of HDOL in the third batch dropped to 12%. The decrease in the catalytic activity with the temperature could be due to the crystallization and aggregation of the RuCoP nanoparticles, which result in a decrease in the number of active sites on the surface of the RuCoP nanoparticles, further leading to a drop in the catalytic activity [38]. After 3 times, the amorphous RuCoP/C gradually changes to crystallization, and the surface area is reduced. The stability of RuCoP/C could be improved in future work. Therefore, the results indicated that the high catalytic activity of RuCoP/C resulted from its rich active sites due to its amorphous phase and small particle size.

## 4. Conclusions

RuCoP/C with low Ru content prepared via the co-precipitation process exhibited the highest selectivity and yield for the hydrogenation reaction of AA to HDOL owing to the electronic effect. The selectivity and yield of HDOL for the optimized RuCoP/C sample was achieved to 80% and 64%, respectively, at 65 bar and 220 °C. The high catalytic performance of RuCoP/C was derived from its small particle size and amorphous state, which provide the more active sites for catalysis. The results indicate that RuCoP with an amorphous structure is a promising catalyst for the conversion of AA to HDOL directly.

## Figures and Tables

**Table 1:** The conversion of AA and selectivity to HDOL on the as-prepared catalysts.

Catalyst	Conversion of AA (%)	Selectivity (%)		
		HDOL	Cyclopentane	Others
No	30	10	10	80
RuP/C	60	16.6	40	43.4
Ru_0.2_Co_7_P_20_/C	60	21	33	46
Ru_0.4_Co_7_P_20_/C	68	33	20	47
Ru_0.6_Co_7_P_20_/C	73	42	28.5	29.5
Ru_0.8_Co_7_P_20_/C	80	70	20	10
Ru_1.0_Co_7_P_20_/C	80	80	11.2	8.8
Ru_1.2_Co_7_P_20_/C	80	80	9.3	10.7
Ru_1.0_Co_3.5_P_20_/C	79.2	67.8	22	12.2
Ru_1.0_Co_7_P_20_/C	80	80	11.2	8.8
Ru_1.0_Co_10.5_P_20_/C	82.1	62.5	18.6	18.9
Ru_1.0_Co_14_P_20_/C	80	62.5	19.8	17.7
Ru_1.0_Co_17.5_P_20_/C	79.6	59	20	21
Ru_1.0_Co_3.5_P_6.7_/C	75	46.6	30	23.4
Ru_1.0_Co_7_P_13.4_/C	80	68.8	25	6.2
Ru_1.0_Co_7_P_20_/C	80	80	11.2	8.8
Ru_1.0_Co_14_P_26.8_/C	82	59.8	20	20.2
Ru_1.0_Co_17.5_P_33.5_/C	82	54.9	25	20.1

## Data Availability

Not applicable.

## Supplementary Materials

The following are available online at https://www.mdpi.com/article/10.3390/ma15228084/s1, Figure S1: Effect of temperature (a) and pressure (b) on AA conversion and HDOL selectivity. Figure S2: XPS survey spectra of all the alloys (a); P 2p XPS spectra of the different metal-doped alloy(b); Ru 3p XPS spectra of RuFeP(c), RuCoP(d), RuNiP (e), RuCuP(f) and RuZnP(g) and P 2p XPS spectra of RuFeP(h), RuCoP(i), RuNiP (j), RuCuP (k) and RuZnP (l). Figure S3: TEM images (a and b) of RuCoP/C. Figure S4: (a) XRD patterns of RuCoP samples heat-treated at different temperatures. TEM images of RuCoP/C-200(b), RuCoP/C-300 (c) and RuCoP/C-400 (d). Figure S5: N2 adsorption and desorption isotherm (e), and BET surface areas of RuCoP/C treated at various temperatures (f). Table S1: Assignments binding energies (BEs) and concentrations of Ru 3p species in as-prepared alloys from Ru 3p XPS spectra.
