# Supplementary material for: Amorphous RuCoP Ultrafine Nanoparticles Supported on Carbon as Efficient Catalysts for Hydrogenation of Adipic Acid to 1,6-Hexanediol
Source: Materials (Basel). 2022 Nov 15;15(22):8084. doi: 10.3390/ma15228084 (PMC9694898; doi:10.3390/ma15228084)
Supplement: Supplementary file 1 [file materials-15-08084-s001.zip › materials-1978119-supplementary.pdf]

materials

*Supplementary Materials*

# Amorphous RuCoP ultrafine nanoparticles supported on carbon as efficient catalysts for hydrogenation of adipic acid to 1,6-hexanediol

**Wei Gong [1], Xuyun Wang [1,*], Shan Ji [2] and Hui Wang [1,*]**

[1] State Key Laboratory Base for Eco-Chemical Engineering, College of Chemical Engineering, Qingdao University of Science and Technology, Qingdao 266042, China
[2] College of Biological, Chemical Science and Chemical Engineering, Jiaxing University, Jiaxing 314001, China
[*] Correspondence: wangxy@qust.edu.cn (X.W.); wangh@qust.edu.cn (H.W.)

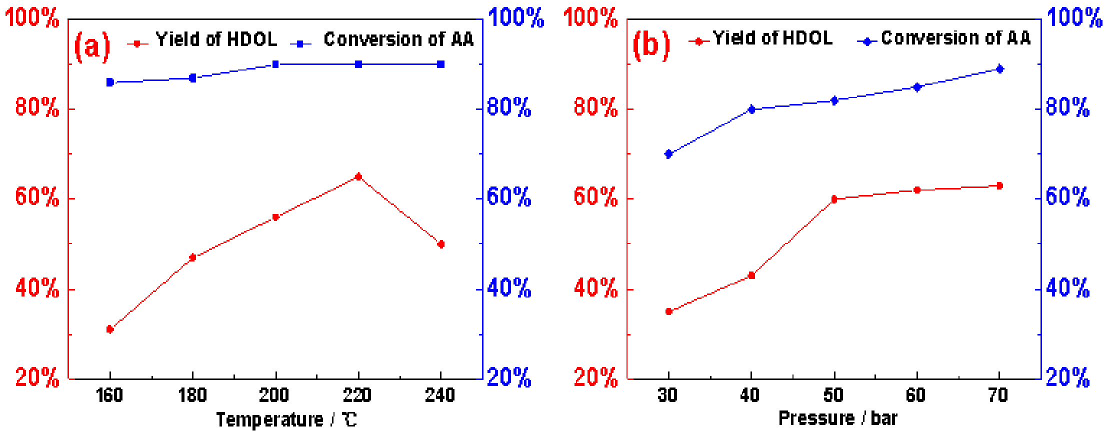


**Figure S1.** Effect of temperature (**a**) and pressure (**b**) on AA conversion and HDOL selectivity.

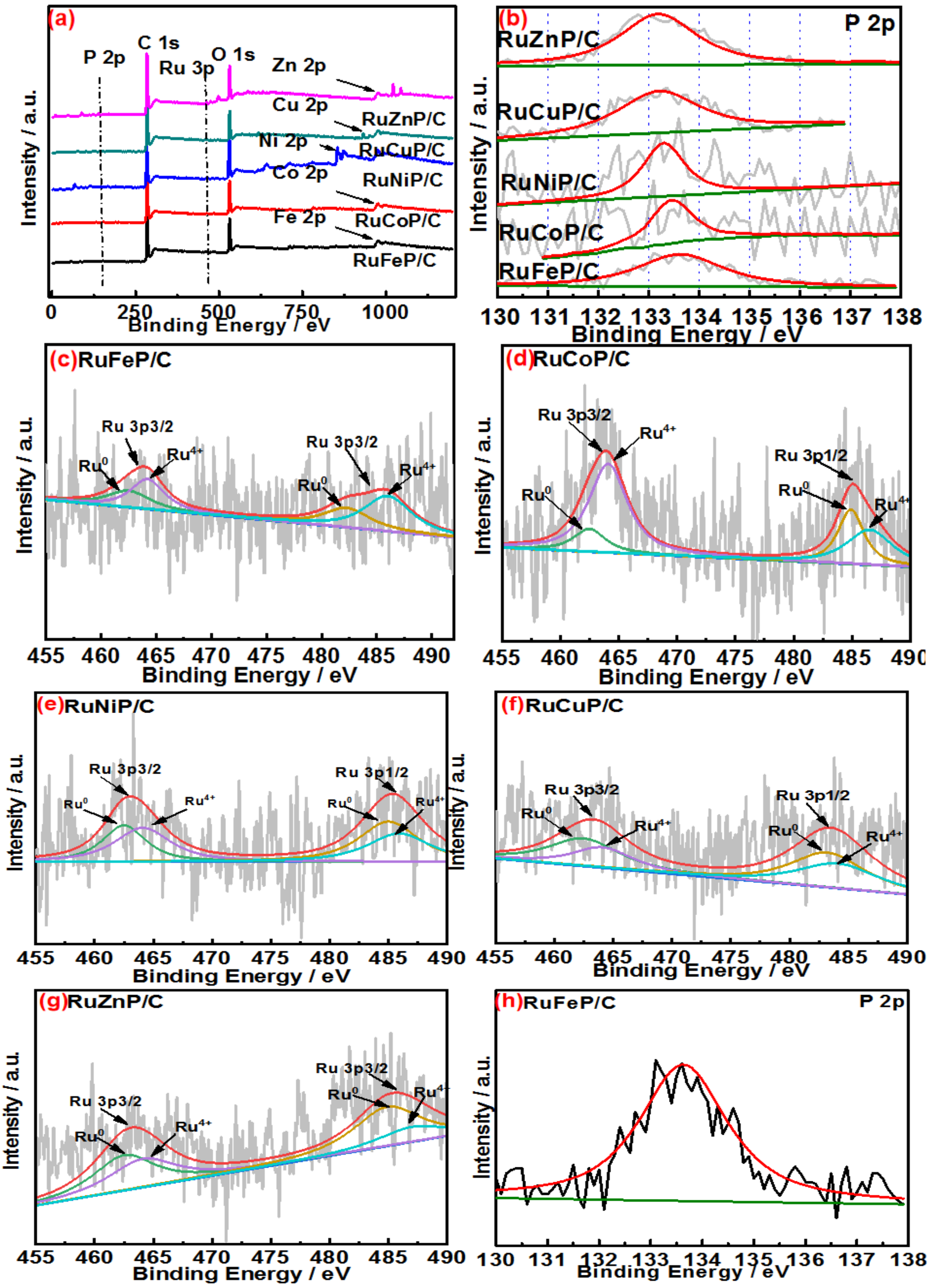

(a)
P 2p
C 1s
Ru 3p
O 1s
Zn 2p
Cu 2p
Ni 2p
Co 2p
Fe 2p
RuZnP/C
RuCuP/C
RuNiP/C
RuCoP/C
RuFeP/C
Intensity / a.u.
Binding Energy / eV
(b)
P 2p
Intensity / a.u.
Binding Energy / eV
(c)RuFeP/C
Ru 3p3/2
Ru0
Ru4+
(d)RuCoP/C
Ru 3p3/2
Ru 3p1/2
(e)RuNiP/C
(f)RuCuP/C
(g)RuZnP/C
(h)RuFeP/C

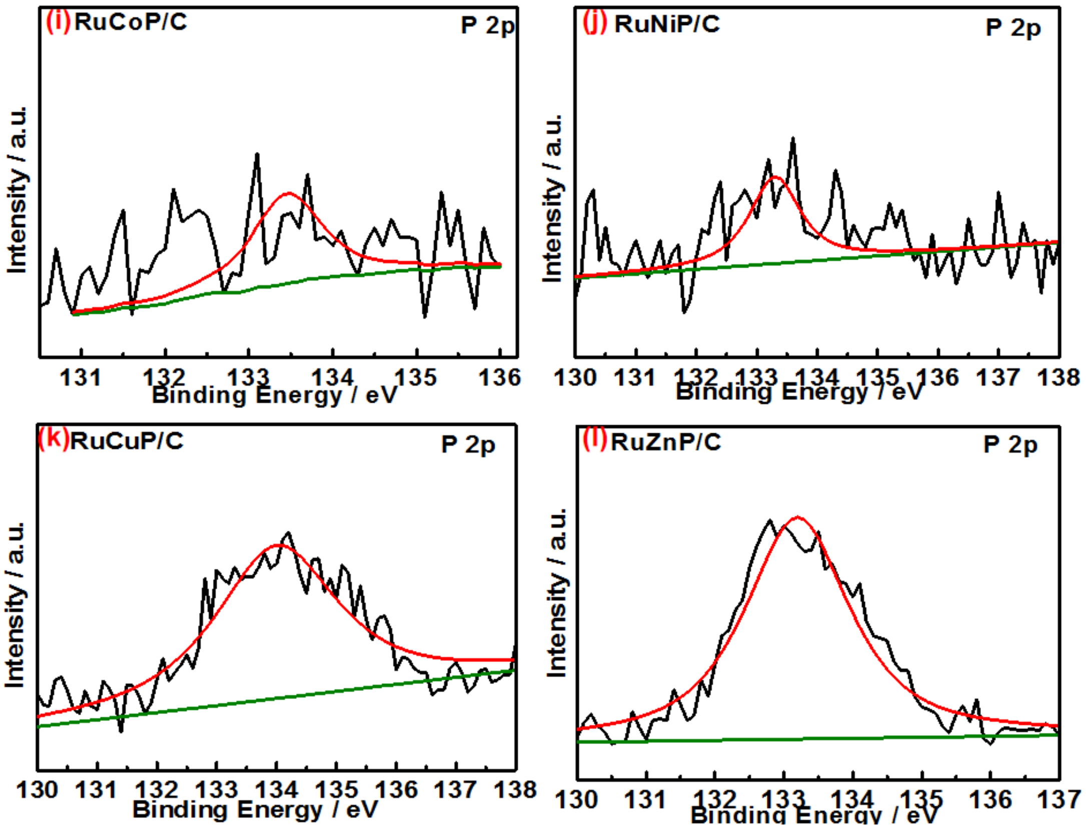


**Figure S2.** XPS survey spectra of all the alloys (**a**); P 2p XPS spectra of the different metal-doped alloy(**b**); Ru 3p XPS spectra of RuFeP(**c**), RuCoP(**d**) , RuNiP (e), RuCuP(**f**) and RuZnP(**g**) and P 2p XPS spectra of RuFeP(**h**) , RuCoP(**i**), RuNiP (**j**), RuCuP (**k**) and RuZnP (**l**).

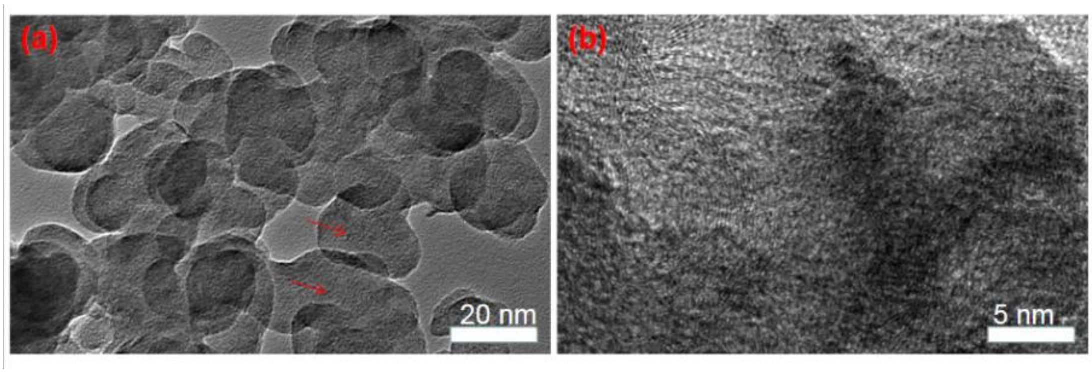


**Figure S3.** TEM images (**a** and **b**) of RuCoP/C.

**Table S1.** Assignments binding energies (BEs) and concentrations of Ru 3p species in as-prepared alloys from Ru 3p XPS spectra.

| Catalyst | Ru 3p | BE[a] | Concentration[b] |
|---|---|---|---|
| RuFeP/C | $Ru^{o}$ | 462.5, 482.5 | 0.42 |
| | $Ru^{4+}$ | 464.5, 486 | 0.58 |
| RuCoP/C | $Ru^{0}$ | 462.5, 485 | 0.53 |
| | $Ru^{4+}$ | 464, 486.5 | 0.47 |
| RuNiP/C | $Ru^{0}$ | 462.5, 485 | 0.49 |
| | $Ru^{4+}$ | 464, 486 | 0.51 |
| RuCuP/C | $Ru^{0}$ | 462.5, 483 | 0.63 |
| | $Ru^{4+}$ | 464, 484 | 0.37 |
| RuZnP/C | $Ru^{0}$ | 462.5, 485 | 0.45 |
| | $Ru^{4+}$ | 464.5, 487.5 | 0.55 |

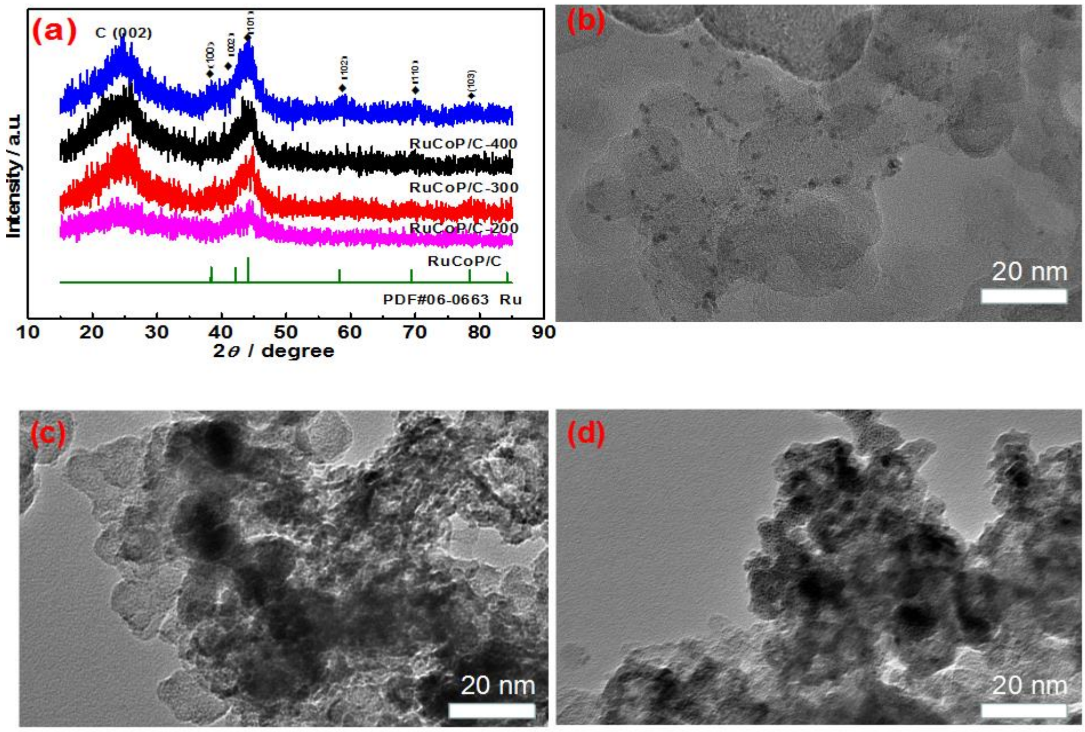


**Figure S4.** (**a**) XRD patterns of RuCoP samples heat-treated at different temperatures. TEM images of RuCoP/C-200(**b**), RuCoP/C-300 (**c**) and RuCoP/C-400 (**d**).

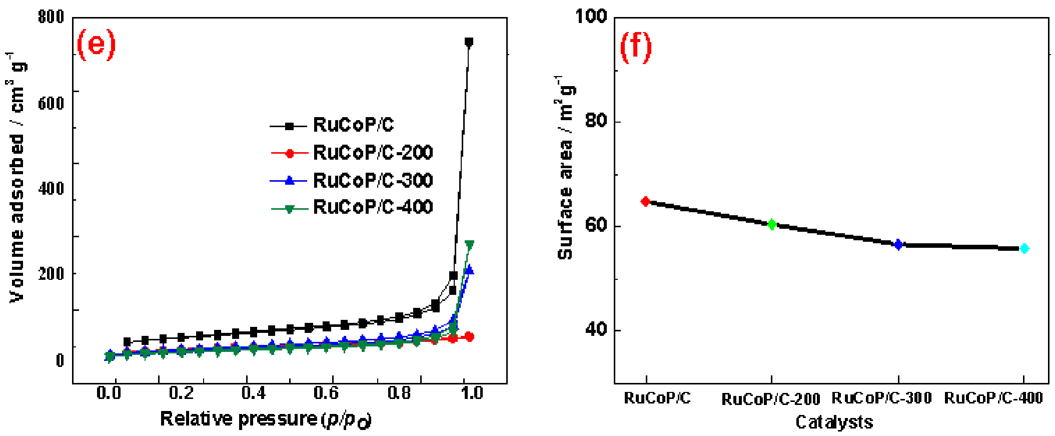


**Figure S5.** $N_2$ adsorption and desorption isotherm (**e**), and BET surface areas of RuCoP/C treated at various temperatures (**f**).
